# Cocaine

**Published:** 1887-06

**Authors:** 


COCAINE.—Dr. Bignon, of Lima, (*American Practitioner and News*,) from a series of experiments on the dog and man, has noted the following results:

1. That cocaine produces only temporary physiological effects in doses from 20 to 50 centigrams when administered by the stomach, on condition of its being absorbed in small divided doses (of five centigrams each hour).

2. It acts principally on the renal secretion in slackening that function, interfering with the elimination of the products of oxidation and thus producing the first symptoms of slight uræmia.

3. In large doses, given at once, it produces anuria, which is followed by grave uræmic accidents, nervous attacks, convulsions, etc.

4. The paralyzed kidney generally regains its function two or three hours after the absorption of the alkaloid; then follows considerable diuresis, which is the more active the longer the duration of the anuria, and relief to the organism.

5. Cocaine is only indirectly toxic when the dose is sufficiently high to prolong the anuria to the point of uræmic poisoning.

6. If the diuresis should quickly remove the toxic phenomena, the general stimulating action continues none the less for a long time afterwards; it lasts about twenty-four hours (in a dose of fifty centigrams to be given in the course of the day). During this time, the phenomena of oxidation continue to go beyond the normal average. In a word, denutrition continues.

